# Changes in hepatic metabolic profile during the evolution of STZ-induced diabetic rats via an ^1^H NMR-based metabonomic investigation

**DOI:** 10.1042/BSR20181379

**Published:** 2019-04-23

**Authors:** Minjiang Chen, Hong Zheng, Min Xu, Liangcai Zhao, Qianqian Zhang, Jingjing Song, Zhongwei Zhao, Siming Lu, Qiaoyou Weng, Xulu Wu, Weibin Yang, Xiaoxi Fan, Hongchang Gao, Jiansong Ji

**Affiliations:** 1 Key Laboratory of Imaging Diagnosis and Minimally Invasive Intervention Research, The Fifth Affiliated Hospital of Wenzhou Medical University/Affiliated Lishui Hospital of Zhejiang University/The Central Hospital of Zhejiang Lishui, Lishui 323000, China; 2 School of Pharmaceutical Sciences, Wenzhou Medical University, Wenzhou 325035, China

**Keywords:** liver, Metabolism, Type 1 diabetes mellitus (T1DM)

## Abstract

**Background:** The present study aimed to explore the changes in the hepatic metabolic profile during the evolution of diabetes mellitus (DM) and verify the key metabolic pathways. **Methods:** Liver samples were collected from diabetic rats induced by streptozotocin (STZ) and rats in the control group at 1, 5, and 9 weeks after STZ administration. Proton nuclear magnetic resonance spectroscopy (^1^H NMR)-based metabolomics was used to examine the metabolic changes during the evolution of DM, and partial least squares-discriminate analysis (PLS-DA) was performed to identify the key metabolites. **Results:** We identified 40 metabolites in the ^1^H NMR spectra, and 11 metabolites were further selected by PLS-DA model. The levels of α-glucose and β-glucose, which are two energy-related metabolites, gradually increased over time in the DM rats, and were significantly greater than those of the control rats at the three-time points. The levels of choline, betaine, and methionine decreased in the DM livers, indicating that the protective function in response to liver injury may be undermined by hyperglycemia. The levels of the other amino acids (leucine, alanine, glycine, tyrosine, and phenylalanine) were significantly less than those of the control group during DM development. **Conclusions:** Our results suggested that the hepatic metabolic pathways of glucose, choline-betaine-methionine, and amino acids were disturbed during the evolution of diabetes, and that choline-betaine-methionine metabolism may play a key role.

## Introduction

Diabetes mellitus (DM) is a complex and chronic metabolic disease characterized by hyperglycemia due to deficiency of insulin secretion or insulin resistance [1]. Epidemiological surveys have shown that approximately 382 million people suffered from DM in 2013 worldwide, and this number will increase to 592 million by 2035 [2]. DM can lead to a series of complications affecting the heart [3], kidneys [4], brain [5], eyes [6], colorectal system [7], and feet [8]. Moreover, studies have shown that DM also causes liver disease, such as hepatic steatosis, nonalcoholic steatohepatitis, fibrosis, and cirrhosis [9–11].

DM has been shown to be associated with liver malfunction. Dey and Swaminathan [12] reported that hyperglycemia can cause dysfunction of hepatocyte mitochondria in both diabetic animals and human patients, a decrease in oxidative phosphorylation, an increase in oxidative stress, and ultrastructural abnormalities. Moreover, a previous study [13] found that hyperglycemia significantly increases the level of glycosylated hemoglobin (HbA1c) and decreases glycogen levels in the livers of type 2 DM (T2DM) rats. Another study [14] also found that hyperglycemia can cause hydroxyl radical-induced apoptosis of hepatocytes in type 1 DM (T1DM) rats. Furthermore, reductions in intracellular nicotinamide adenine dinucleotide (NAD) and adenosine triphosphate (ATP) were observed in the liver of STZ-induced diabetic rats [15]. The liver is the major metabolic organ for maintaining whole-body metabolic homeostasis [16], but the mechanism of DM-induced metabolic disturbance in the liver remains not fully understood.

Metabolomics is carried out to profile all low molecular weight metabolites in biological samples in the context of a specific condition, such as diseases or drug interventions. Our previous study identified metabolic changes in rats and mice with diabetic nephropathy [17,18] and neuron–astrocyte metabolic cooperation in db/db mice [19]. Our current study examined the metabolic changes in the livers of STZ-induced diabetic rats at 1, 5, and 9 weeks using a proton nuclear magnetic resonance spectroscopy (^1^H NMR)-based metabolomic approach, to explore the underlying mechanisms of DM-induced metabolic disorders in rat livers.

## Results

### The histologic features of rat livers

**Figure 1:**
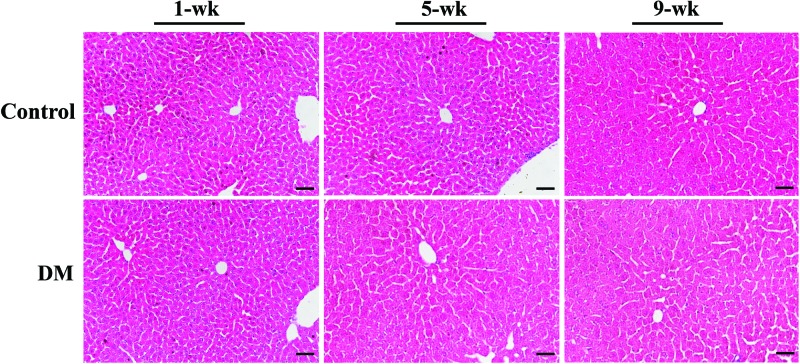
Pathological changes in diabetic livers The representative microscopic photographs of the control and diabetic livers at different time points (1-, 5-, and 9-week) stained with HE (400×); scale bars represented 25 μm.

The typical histologic appearances of livers by Hematoxylin–Eosin (H&E) staining are presented in Figure 1. Compared with the control liver, no characteristic changes were found in the diabetic liver at 1st, 5th, and 9th weeks, and were characterized with a normal central vein, normal hepatocytes, no fatty changes and mild RBC infiltrations, which were similar to the previous study [20].

### ^1^H NMR spectral analysis

**Figure 2:**
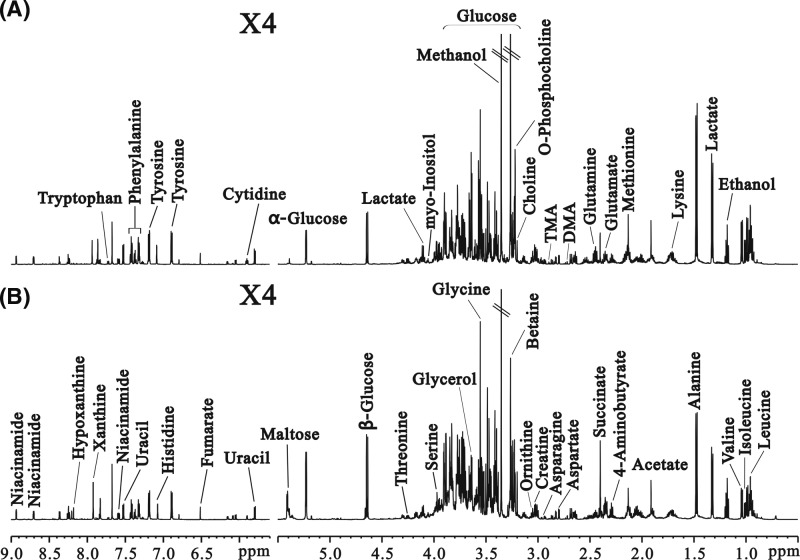
^1^H NMR spectra of diabetic and control livers Representative 600 MHz 1D ^1^H NMR spectra of liver extracts obtained from control rat (**A**) and diabetic rats at 9 weeks (**B**). Forty different endogenous metabolites were identified based on the ^1^H NMR spectra of the liver extracts.

Representative ^1^H NMR spectra of liver samples obtained from control (A) and diabetic (B) rats are shown in Figure 2. The metabolite resonance signals were identified according to our previous work [21] and Chenomx NMR suite 7.0 (Chenomx Inc., Edmonton, Canada). Based on the ^1^H NMR spectra of liver extracts, 40 endogenous metabolites were identified and the concentration was measured simultaneously (Figure 2). These metabolites had a median coefficient of variation (CV) of 9.4% relative to the quality control (QC) samples, ranging from 1.7 to 14.8%, and all the metabolites had CV values < 15%, which indicated their reproducibility.

### Metabolomic analysis

**Figure 3:**
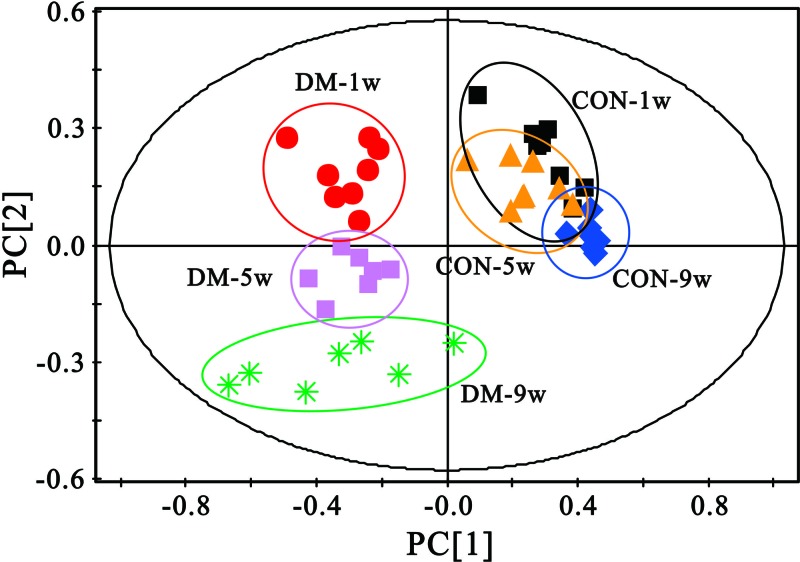
PLS-DA score plots of the six groups, performed on all the samples, and the diabetic samples were scattered, whereas the control samples were clustered together The two groups were clearly discriminated along the direction of PC1. In addition, the diabetic rats at different time points were also clearly separated along the direction of PC2 among the three time points.

**Figure 4:**
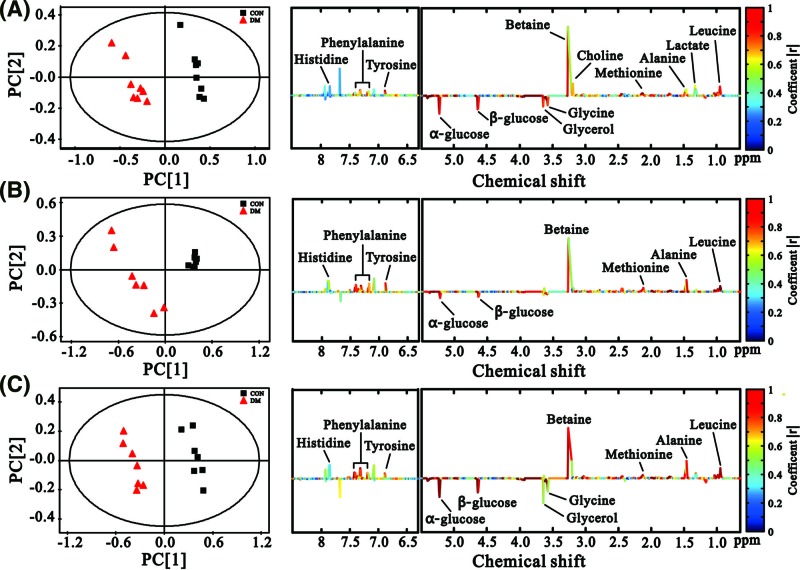
PLS-DA score plots and matched loading plots of the disease effect from NMR-based liver metabolomics of T1DM and control rats From top to bottom: results from rats at 1 (**A**), 5 (**B**), and 9 (**C**) weeks, respectively. Peaks in the positive direction indicate metabolites that are more abundant in the control group than DM group (↑CON). Consequently, metabolites that are more abundant in the DM group are presented as peaks in the negative direction (↓DM).

To explore the metabolic profiles of diabetic livers at different evolutionary stages, partial least squares-discriminate analysis (PLS-DA) was performed based on the ^1^H NMR spectra of the 1-, 5- and 9-week diabetic rats as well as their age-matched controls. Figure 3 shows all of the samples, and the diabetic samples were scattered, whereas the controls were clustered together, and the two groups showed clear discrimination along the direction of principal component (PC) 1. In addition, the diabetic rats were also clearly separated along the direction of PC2 among the three time points. This finding indicates that the metabolic alterations in the diabetic state were more obvious than those in the control state; therefore, age-related factors may be less important compared with diabetes-related causes in the present study. Figure 4 shows that the diabetic rats and their age-matched controls were clearly discriminated along the direction of PC1 at all time points in the score plots (left). The parameters R^2^X, R^2^Y, and Q^2^ for the PLS-DA models indicated that the established models were robust and credible (Table 1). The corresponding loading plots of the PLS-DA models were used to investigate the contribution of different metabolites during the process, and the colors indicate the significance of classification from the correlation matrix. The results indicated that the separation ascribed to the metabolites that have higher correlation (|r| > 0.6, according to the previous study [22]), included leucine, alanine, methionine, choline, betaine, glycine, glycerol, α-glucose, β-glucose, tyrosine, phenylalanine etc.

**Table 1:** The parameters of PLS-DA model

Time	R^2^X	R^2^Y	Q^2^
1 week	0.72	0.983	0.985
5 weeks	0.786	0.979	0.957
9 weeks	0.799	0.987	0.972

**Figure 5:**
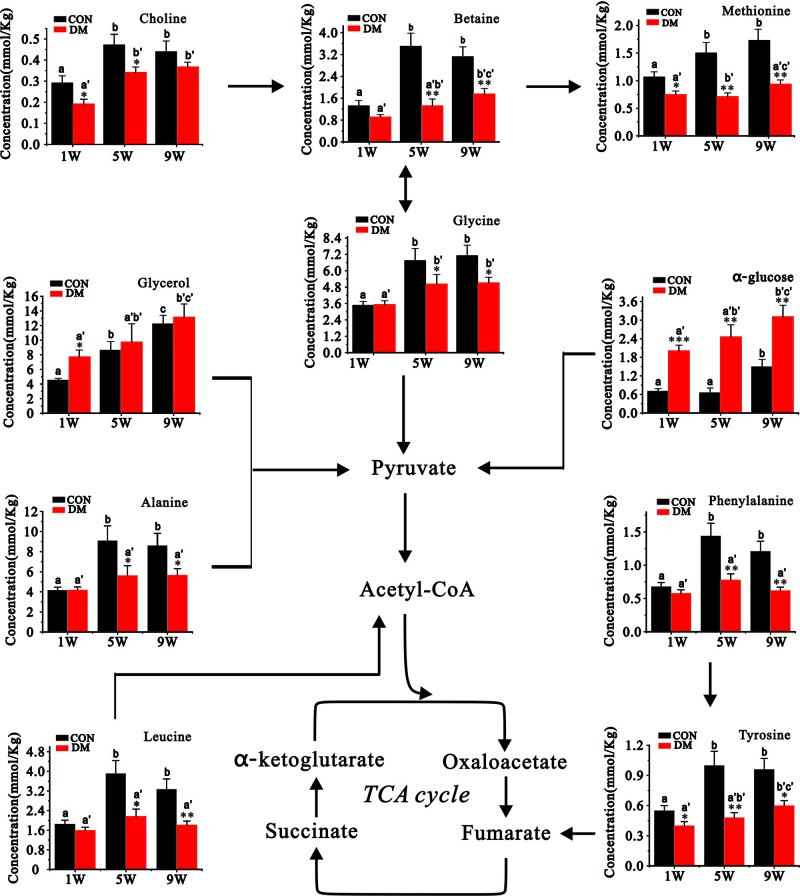
The key metabolites and metabolic pathways are shown, and glucose metabolism, choline-betaine-methionine metabolism, and amino acid metabolism were involved (**A**–**C**) indicate that in a row without a common letter differ for comparisons between the DM and control groups at the same time point, *P*<0.05; (A–C) indicate that in a row without a common letter differ for comparisons compared among the same groups (control groups or DM groups) at different time points.

The concentrations of metabolites in the livers of the DM rats and the rats in the control groups are shown in Table 2, and Figure 5 shows the characteristic changes of metabolites identified by the PLS-DA model. We found that the concentration of α-glucose and β-glucose in rat livers with DM increased significantly over the course of diabetes development and was obviously higher compared with the control group at the specific time point, which was consistent with the development of diabetes. The concentration of glycerol, which can be converted into glucose as an energy supply source, was higher at the 1-week time point after the onset of diabetes (1 week), but there were no differences at the 5th and 9th weeks. Choline, an important component of the cell membrane, was significantly lower in the DM livers at the 1st and 5th weeks, but there was no difference at the 9th week. The content of betaine, an important molecule involved in the process of methylation, which can be converted into methionine and subsequently to methionine metabolism [23], increased significantly in the livers of diabetic rats, but the levels in the livers of control rats were fluctuating, and the levels in the control rats at 5th and 9th weeks were significantly higher than those in the diabetic rats.

**Table 2:** Comparison of hepatic metabolites levels between the DM and control groups at 1-, 5-, and 9-week

Metabolites	1 Week		5 Week		9 Week	
	CON (mmol/kg)	DM (mmol/kg)	CON (mmol/kg)	DM (mmol/kg)	CON (mmol/kg)	DM (mmol/kg)
Valine	1.57 ± 0.13	1.33 ± 0.09	3.40 ± 0.45	1.91 ± 0.26^1^	2.83 ± 0.36	1.50 ± 0.12^2^
Isoleucine	0.91 ± 0.08	0.79 ± 0.06	2.03 ± 0.27	1.14 ± 0.15^1^	1.62 ± 0.21	0.90 ± 0.07^2^
Leucine	1.85 ± 0.16	1.60 ± 0.12	3.92 ± 0.52	2.17 ± 0.29^1^	3.28 ± 0.41	1.82 ± 0.15^2^
Ethanol	0.42 ± ± 0.05	1.25 ± 0.17^2^	0.39 ± 0.05	0.96 ± 0.25^1^	1.20 ± 0.18	1.98 ± 0.29^1^
Lactate	3.51 ± 0.20	3.05 ± 0.23	3.82 ± 0.52	2.76 ± 0.39	6.02 ± 0.70	3.98 ± 0.47^1^
Alanine	4.16 ± 0.30	4.19 ± 0.30	9.12 ± 1.45	5.63 ± 0.97^1^	8.63 ± 1.19	5.67 ± 0.64^1^
Lysine	3.82 ± 0.34	3.37 ± 0.24	8.03 ± 1.05	4.54 ± 0.64^1^	6.87 ± 0.89	3.89 ± 0.33^1^
Acetate	0.51 ± ± 0.05	0.67 ± 0.04^1^	1.07 ± 0.15	0.87 ± 0.16	1.13 ± 0.14	0.93 ± 0.11
Methionine	1.07 ± 0.09	0.75 ± 0.06^1^	1.50 ± 0.19	0.71 ± 0.07^2^	1.74 ± ± 0.19	0.94 ± 0.08^2^
4-Aminobutyrate	1.30 ± 0.14	1.69 ± 0.15	3.36 ± 0.51	2.82 ± 0.53	2.43 ± 0.37	1.94 ± 0.18
Glutamate	2.68 ± 0.24	3.03 ± 0.30	4.47 ± 0.49	3.13 ± 0.13^1^	4.81 ± 0.67	3.87 ± 0.23
Succinate	2.15 ± 0.13	2.37 ± 0.26	2.54 ± 0.38	3.07 ± 0.23	3.78 ± 0.41	4.30 ± 0.16
Glutamine	2.42 ± 0.22	1.29 ± 0.13^3^	2.29 ± 0.46	0.61 ± 0.10^2^	3.88 ± 0.41	2.00 ± 0.21^3^
Aspartate	1.01 ± 0.10	0.98 ± 0.06	2.00 ± 0.28	1.21 ± 0.17^1^	1.78 ± 0.25	1.14 ± 0.10^1^
Dimethylamine	0.11 ± 0.01	0.12 ± 0.01	0.17 ± 0.02	0.14 ± 0.02	0.19 ± 0.03	0.17 ± 0.01
Asparagine	0.53 ± 0.03	0.44 ± 0.05	0.58 ± 0.08	0.21 ± 0.04^3^	0.79 ± 0.08	0.45 ± 0.05^2^
Trimethylamine	0.04 ± 0.00	0.04 ± 0.00	0.06 ± 0.01	0.03 ± 0.01^1^	0.07 ± 0.01	0.05 ± 0.01
Creatine	0.45 ± 0.05	0.47 ± 0.04	0.91 ± 0.12	0.72 ± 0.15	0.92 ± 0.12	0.49 ± 0.05^1^
Ornithine	1.35 ± 0.12	1.19 ± 0.08	2.66 ± 0.35	1.59 ± 0.26^1^	2.44 ± 0.31	1.44 ± 0.12^1^
Choline	0.29 ± 0.03	0.19 ± 0.02^1^	0.47 ± 0.05	0.34 ± 0.03^1^	0.44 ± 0.05	0.37 ± 0.02
*o*-Phosphocholine	0.56 ± 0.04	0.59 ± 0.05	0.92 ± 0.14	0.72 ± 0.16	1.19 ± 0.13	0.60 ± 0.08^2^
Betaine	1.32 ± 0.20	0.91 ± 0.09	3.50 ± 0.48	1.32 ± 0.25^2^	3.12 ± 0.36	1.75 ± 0.21^2^
Glycine	3.46 ± 0.29	3.52 ± 0.28	6.74 ± 0.89	5.02 ± 0.71^1^	7.10 ± 0.77	5.11 ± 0.41^1^
Glycerol	4.51 ± 0.27	7.71 ± 0.94^1^	8.61 ± 1.20	9.74 ± 2.49	12.22 ± 1.18	13.14 ± 1.81
*myo*-Inositol	0.54 ± 0.04	0.56 ± 0.05	0.94 ± 0.12	0.66 ± 0.06^1^	0.92 ± 0.10	0.86 ± 0.07
Serine	5.33 ± 0.38	9.06 ± 0.99^2^	8.80 ± 1.19	11.70 ± 2.57	9.96 ± 1.20	13.24 ± 1.70
Threonine	1.86 ± 0.14	1.67 ± 0.13	3.32 ± 0.45	1.95 ± 0.26^1^	3.34 ± 0.40	2.05 ± 0.18^1^
α-Glucose	0.70 ± 0.09	2.01 ± 0.18^3^	0.65 ± 0.16	2.46 ± 0.39^2^	1.49 ± 0.24	3.11 ± 0.37^2^
β-Glucose	0.77 ± 0.10	1.86 ± 0.15^3^	0.73 ± 0.18	2.29 ± 0.35^2^	1.60 ± 0.26	2.76 ± 0.30^1^
Uracil	0.31 ± 0.03	0.31 ± 0.02	0.49 ± 0.07	0.35 ± 0.05	0.60 ± 0.09	0.39 ± 0.05
Cytidine	0.06 ± 0.01	0.07 ± 0.01	0.16 ± 0.02	0.14 ± 0.03	0.11 ± 0.01	0.09 ± 0.01
Fumarate	0.09 ± 0.01	0.18 ± 0.02^3^	0.19 ± 0.03	0.18 ± 0.05	0.22 ± 0.04	0.18 ± 0.03
Tyrosine	0.55 ± 0.05	0.40 ± 0.04^1^	1.00 ± 0.14	0.48 ± 0.05^2^	0.96 ± 0.11	0.60 ± 0.05^1^
Histidine	0.56 ± 0.06	0.48 ± 0.04	0.86 ± 0.11	0.35 ± 0.02^2^	1.06 ± 0.15	0.58 ± 0.05^1^
Phenylalanine	0.68 ± 0.06	0.58 ± 0.05	1.44 ± 0.19	0.78 ± 0.09^2^	1.21 ± 0.15	0.62 ± 0.05^2^
Tryptophan	0.14 ± 0.01	0.12 ± 0.01	0.30 ± 0.04	0.16 ± 0.02^3^	0.25 ± 0.03	0.13 ± 0.01^2^
Hypoxanthine	0.11 ± 0.02	0.12 ± 0.02	0.05 ± 0.01	0.05 ± 0.01	0.12 ± 0.01	0.20 ± 0.02^2^
Niacinamide	0.31 ± 0.03	0.35 ± 0.02	0.38 ± 0.05	0.33 ± 0.05	0.60 ± 0.07	0.52 ± 0.04
Xanthine	0.13 ± 0.01	0.12 ± 0.02	0.09 ± 0.01	0.10 ± 0.00	0.19 ± 0.01	0.17 ± 0.01
Maltose	0.18 ± 0.05	4.48 ± 0.77^3^	0.13 ± 0.03	6.24 ± 2.71^1^	0.44 ± 0.12	7.49 ± 1.23^2^

^1^*P*<0.05, ^2^*P*<0.01, ^3^*P*<0.001 on comparison between DM rats and CON rats at the same time-points.

The amino acid metabolites between the two groups, such as leucine, methionine, glycine, alanine, tyrosine, and phenylalanine, were also significantly different. The content of leucine, one of the branched-chain amino acids (BCAAs), was constantly lower in the livers of rats with DM at the 5th and 9th weeks, but the levels in the control livers at the 5th and 9th weeks were significantly greater than that at 1st week. Glycine in the DM livers and control livers was all gradually increased during the evolution of diabetes, methionine was also increased gradually in control livers, and significantly elevated in the DM livers at 9th week, both the metabolites in DM group were significantly lower than those in the control group. Alanine and phenylalanine were significantly lower in the livers of rats with diabetes at the 5th and 9th weeks, but there was no difference at 1st week, compared with the increasing levels of these two metabolites in the control group during the period of observation, the levels in DM group remained unchanged. The levels of tyrosine in the livers of rats in the control and the DM groups increased over time, but the increased level in the control group was significantly greater than that in the DM group, which led to a significant difference between the two groups.

## Materials and methods

### Animals

Forty-four male Sprague–Dawley rats weighing 160–180 g were purchased from Shanghai SLAC Laboratory Animal Co. Ltd. Throughout the entire period of experimentation, all rats were kept in a specific pathogen free (SPF) colony in an environment with regulated temperature, humidity, and a 12/12-h light–dark cycle. The rats were fed with certified standard rat chow and tap water *ad libitum* and were acclimated for 1 week prior to receiving treatments. The protocol for the animal experiments was approved by the Institutional Animal Care and Use Committee of the Fifth Affiliated Hospital of Wenzhou Medical University, and the related procedures were conducted at Experimental Animal Center of Wenzhou Medical University. All animals received care in accordance with the ‘Guide for the Care and Use of Laboratory Animals’.

### Experimental design and sample collection

Rats were randomly assigned to six groups (*n*=7–8 rats per group), including rats with 1-week diabetes induced by STZ (*n*=8), 5-week diabetes (*n*=7), 9-week diabetes (*n*=7), and three age-matched control groups. After overnight fasting, rats assigned to the diabetic groups received an intraperitoneal injection of streptozotocin (STZ, Sigma–Aldrich), which was freshly prepared in citrate buffer (0.1 M, pH 4.5), at a single dose of 60 mg/kg body weight. The rats in the control group received an injection of the same volume of sodium citrate. Three days after the treatment, the level of blood glucose was measured using a tail nick and glucometer (B. Braun, Melsungen AG, Germany; detection range 0.6–33.3 mmol/l). Blood glucose levels greater than 16.70 mmol/l were defined as the diagnostic criteria for diabetes in rats (*n*=22).

We found that the blood sugar levels of all STZ-treated rats reached 16.7 mM on day 3 after the administration of STZ and maintained the same level for 1 week, which met the criteria for diagnosing diabetes [24]. As expected, the diabetic animals exhibited many symptoms commonly associated with diabetes (e.g. polyuria, polydipsia, and diarrhea), and the body weight decreased over the entire period of experimentation (data not shown).

At each time point, after overnight fasting (12 h) but with free access to water, the rats were killed by decapitation and the livers were dissected, which were cut into two pieces, one piece was fixed in 4% paraformaldehyde, embedded in paraffin for further H&E staining, the other one snap-frozen in liquid nitrogen, and stored at −80°C for further NMR spectroscopy analysis.

### Liver metabolite extraction and preparation of samples

The frozen liver tissue was weighed and placed into a centrifuge tube containing ice-cold methanol (4 ml/g) and distilled water (0.85 ml/g). The tissue was homogenized at 4°C after being thawed and mixed using a vortex mixer. Chloroform (2 ml/g) and distilled water (2 ml/g) were added to the tube followed by vortexing. After 15 min of incubation on ice, the homogenate was centrifuged at 1000×***g*** for 15 min at 4°C. The supernatant was collected and lyophilized for 24 h. The obtained aqueous extracts were dissolved in 550 μl of 0.4 mmol/l trimethylsilyl-propionic-2,2,3,3d4-acid (TSP) in D_2_O, vortexed, and then centrifuged at 10000×***g*** for 10 min at 4°C. A total of 500 μl of supernatant was transferred to a 5-mm NMR tube for NMR spectroscopy analysis. All samples were randomized during sample preparation. A pooled sample from all the samples was extracted using the same procedure as described above, which was used as the QC sample and was analyzed once for every five to ten study samples.

### Acquisition of ^1^H NMR data

The ^1^H NMR spectroscopy analysis was performed on a Bruker Advance III 600 spectrometer at 298 K by a standard single-pulse sequence with water presaturation (‘zgpr’, Bruker BioSpin, Gmbh, Rheinstetten, Germany) using a 90° flip angle, 12 kHz spectral width, 32 K data points, 2.65 s acquisition time, 8 s relaxation delay. The total number of scans was 128. The spectra were zero-filled to 64 K, and an exponential line-broadening function of 0.3 Hz was applied to the free induction decay prior to Fourier transformation. All spectra were manually phased and baseline-corrected with reference to the chemical shift of the methyl peak of lactate (CH3, 1.33 ppm) using Topspin (v2.1 pl4, Bruker Biospin, Germany) software.

### Data processing of NMR spectra

All NMR spectra (−1 to 10.0 ppm) were divided into integral regions with equal widths of 0.01 and 0.0015 ppm (2.4 Hz) by using Bruker Topspin 2.1 software (v2.1 pl4, Bruker Biospin, Germany). Each segment consisted of the integral of the associated NMR region except for δ4.80–4.69 (containing the residual peak from the suppressed water resonance), which was set to zero integral in the analysis. Due to the presence of conspicuous metabolite of glucose, the resonance regions of glucose in all spectra were excluded [25,26], except for the regions of α-glucose and β-glucose. To minimize the exogenous metabolite effect, methanol signals from 3.36 to 3.34 were also set to the zero integral.

### Multivariate pattern recognition analysis

Multivariate analysis of the metabolomics data was performed using PLS-DA, which is capable of reducing the dimensionality of the data while maximizing the variance between the dependent and exploratory variables, and providing the most efficient 2D representation of the information. The parameters R^2^X, R^2^Y, and Q^2^ were used to evaluate the models. R^2^X and R^2^Y indicate the goodness-of-fit and Q2 indicates the predictive ability. In our study, the PLS-DA model was used to define the discrimination of metabolites between the control and DM groups at different time points using SIMCA 13.0 software (Umetrics, Umeå, Sweden). In addition, the corresponding loading plots were assessed by the absolute value of the correlation coefficient, |*r*|, which was used to identify the metabolites that mostly contributed to the discrimination between two groups at the three-time points. The loading plots were analyzed using MATLAB software (R2012a, MathWorks Inc., Natick, MA, U.S.A.).

### Statistical analysis

TSP was used as the internal reference and the relative concentrations of the metabolites were determined based on the spectra and normalized to the weight of the freeze-dried metabolite mixture. Statistical analysis was carried out using SPSS software (version 16.0, SPSS). Independent samples *t* test was used to compare the relative signal integrals between the two groups, and one-way analysis of variance (ANOVA) and Tukey’s HSD post hoc testing were used to analyze the differences between the different age groups. A *P*-value of 0.05 was considered statistically significant.

## Discussion

T1DM, a chronic metabolic disorder, has been shown to be associated with liver dysfunction [27–30], but the underlying metabolic mechanisms remain poorly understood. Our current study investigated the metabolic characteristics of the liver in STZ-induced diabetic rats at 1, 5, and 9 weeks using NMR-based metabolomics. No characteristic changes of histologic features were found in the diabetic liver at 1st, 5th, and 9th weeks, but the metabolomic results demonstrated the metabolic changes of glucose, lipid, and amino acid in the liver during the progression of T1DM.

## Glucose metabolism in the liver during T1DM progression

The liver, which is one of the major metabolic organs that maintains the systemic glucose homeostasis and gluconeogenesis, plays a critical role in maintaining euglycemia in the liver. Abnormally increased hepatic gluconeogenesis, which contributes to chronic hyperglycemia, has been recognized during diabetes [31]. Chronic hyperglycemia, the major cause of diabetes-related complications [32], has also been shown to be involved in the pathogenesis of DM-induced inflammation in the liver [33], and the up-regulation of tumor necrosis factor-α, interleukin-6, and inducible nitric oxide synthase (iNOS) has been observed in both animal models and patients with diabetes [34]. Our study demonstrated that the concentrations of hepatic glucose in rat livers with DM were significantly greater than those in the control groups at the three different time points and that concentration was constantly increasing over the course of DM evolution, with the results showing that the concentration of α-glucose in the livers of the diabetic rats was 2.09-times greater than that of the control group at the 9th week and had increased by 54.7% compared wit hthat at the 1st week. The concentration of β-glucose was 1.7 times greater than that of the control group at the 9th week, which had increased by 48% compared with that at the 1st week. These results indicated that homeostasis of hepatic glucose was disturbed during the development of T1DM.

Glucose metabolism provides most energy to maintain the function of the body through the process of aerobic oxidation and glycolysis. The tricarboxylic acid (TCA) cycle involves the aerobic oxidation of glucose and is an important pathway for producing energy [35]. Our study identified the abnormal metabolism of succinate and fumarate, which are the two intermediates of the TCA cycle in the livers of diabetic rats. The concentration of fumarate in the livers of diabetic rats at the 1st week was significantly greater than that in the control group, but the differences between the two metabolites at the three time points were not significant. No significant difference in lactate, which is a product of glycolysis [36], at the 1st and 5th weeks was observed between the diabetic livers and the control livers, while the concentration of lactate in the DM group was significantly less than that of the control group at the 9th week and was only 66.1% of the level in the control group. These results suggested that the TCA cycle was affected by DM, but the glycolytic activity was weakened during the development of DM, indicating that energy metabolism of diabetic livers was in disorder.

## Liver choline-betaine-methionine metabolism during T1DM development

Choline, which is one of the products of lipid metabolism, is important for maintaining the integrity of cellular structure, methyl (one-carbon) metabolism, and lipid/cholesterol transport and metabolism [37,38]. Choline can be converted into betaine by choline oxidase. Previous have studies demonstrated that the deficiency of choline is closely linked with liver diseases, such as liver cirrhosis [39] and hepatic steatosis [40]. The present study showed that the level of choline in the DM groups was significantly less than that in the control groups at the 1st and 5th weeks, showing that the hyperglycemic state may affect the function of livers in the DM condition.

Betaine, which is a methyl provider, has a close relationship with liver disease [41]. A previous study showed that the administration of betaine can reverse hepatic fat accumulation and injury in mice by improving the function of adipose tissue [42]. Another study also confirmed that betaine could markedly attenuate hepatotoxicity and fibrosis induced by dimethylnitrosamine, and protect the liver from fibrogenesis by maintaining the cellular antioxidant capacity [43]. The previous study also showed that betaine could function as a protective factor against liver injury induced by alcohol, which was facilitated by improving impaired sulfur-containing amino acid metabolism [44]. Our study showed that the betaine level in the livers of diabetic rats significantly decreased compared with that of the corresponding rats in the control group, which may contribute to hepatic injury.

Methionine, which is an essential amino acid, is the precursor of S-adenosylmethionine (SAM) and the abnormal metabolism of methionine is associated with liver diseases [45]. The disturbed metabolism of methionine induced by folate deficiency had proven to worsen liver injury in ethanol-fed micropigs [46], and the altered metabolism of methionine also had demonstrated that could promote hepatocellular apoptosis and proliferation [47]. A previous study also showed that the decreased methionine could contribute to alcoholic hepatitis in humans [48]. Previous studies have also demonstrated that the relationship between methionine and liver diseases is mediated by SAM, because methionine is the universal methyl provider for the methylation of DNA, RNA, and proteins [49]. Our study showed that the levels of methionine were significantly lower in the DM groups compared with the control groups, indicating that the pathway of methionine metabolism was disturbed in the evolution of T1DM and predisposed the liver to any injuries.

## Liver amino acid metabolism during DM development

The disturbance of amino acid metabolism is common in diseased livers because the liver is the major organ for amino acid conversion. Our study showed the disordered metabolism of amino acid during DM development, manifesting that the levels of leucine, alanine, phenylalanine, tyrosine, and glycine in STZ-treated rats at the 5th and 9th weeks were significantly less than those in the corresponding control group.

Substantial evidence has confirmed that the supplementation of leucine, a branched-chain ketogenic amino acid, could decrease the incidence of complications of liver disease [50], which indirectly indicates that leucine could improve liver function and act in a protective role against liver disease. A previous study showed that leucine could retard hepatocarcinogenesis, improve immune function, lessen oxidative stress and, in turn, reduce the occurrence of complications in cirrhotic patients [51]. Another study speculated that leucine could be used as a nutritional supplement to improve the clinical outcome of chronic liver disease [52]. Our study showed that the level of leucine in the rat livers with DM was maintained at a constantly lower level than that in the control livers at the 5th and 9th weeks, which may result from the increased consumption of leucine acting in a protective role against hepatic injury caused by hyperglycemia.

Alanine is the major precursor of gluconeogenesis in the human body and can be directly converted into pyruvate by alanine aminotransferase. A previous study showed that the conversion of alanine into glucose was significantly higher in livers affected by DM [53]. Our study showed that the level of alanine in the rat livers with DM was significantly less than that in the rat livers of the control groups at the 5th and 9th weeks, which may be attributed to the increased gluconeogenesis of diabetic livers. The liver is the principal site of the hydroxylation of phenylalanine to tyrosine and further tyrosine degradation. Previous studies have shown that metabolic disturbance of phenylalanine and tyrosine is responsible for many clinical disorders of livers [54,55]. In our study, the levels of phenylalanine and tyrosine in the DM groups were significantly less than those in the DM groups at the 5th and 9th weeks, indicating that phenylalanine and tyrosine metabolism were impaired during DM development.

Previous studies have shown that glycine, which is a nonessential amino acid can protect the liver from hypoxia, ischemia, and ATP depletion-induced injury [56,57]. Another study also showed that glycine can significantly reduce oxidative stress and enhance the protective function of enzymic and nonenzymic antioxidants against alcohol-induced liver damage [58]. Glycine also had proved to reduce the systemic inflammatory response and maintain the production of cellular energy to attenuate hepatic ischemia–reperfusion injury [59]. In our study, the level of glycine in the rat livers with DM was significantly less than that in the control livers at the 5th and 9th weeks, indicating that glycine may be overconsumed to protect against hyperglycemic toxicity in the DM liver.

In current study, the changes of multiple metabolites in diabetic livers showed stage-specific feature. The acetate, glycerol, and serine in the diabetic liver were significantly greater than those in the control group at 1 week, and there was no significant change at 5 and 9 weeks, which may be related to the liver toxicity of STZ [60]. The levels of glutamate, trimethylamine, and *myo*-Inositol in diabetic liver were significantly less compared with the control group at 5 weeks, and there was no significant difference at 1 and 9 weeks, and the levels of lactate, creatine, *o*-phosphocholine, and hypoxanthine in diabetic lives also only showed significant changes at 9 weeks, which may be highly correlated with the stage of diabetes progression and required further exploration and validation.

Our study had some limitations. First, we observed the changed metabolic profile of rat livers at three different time points (1, 5, and 9 weeks after the initiation of diabetes), but we did not extend the follow-up period to evaluate the long-term effect. Second, we showed that the pathway of methionine metabolism was significantly changed in diabetic liver, but a combination analysis with genomics or proteomics should be performed to better understand the disease progression.

In summary, ^1^H NMR-based metabolomics has great value for the identification and quantitation of diabetes-related abnormal metabolites in the liver, which may provide insight into the pathogenesis of diabetic livers from the perspective of hepatic metabolomics. NMR spectroscopy-based liver metabolomics showed the spectrum of metabolite changes in a rat model with T1DM, including the remarkable content changes of leucine, alanine, methionine, choline, betaine, glycine, glycerol, α-glucose, β-glucose, tyrosine, and phenylalanine, in the livers with DM, indicating the potential of further investigation of the abnormalities in the metabolic pathways of glucose, choline-betaine-methionine, and amino acids. Our study showed that the levels of protective factors significantly decreased during the progression of diabetes, including glycine, choline, betaine, and methionine, which are the metabolites in the choline-betaine-methionine metabolic pathway. Further investigations are needed to explore the molecular mechanisms involved in the pathogenesis of diabetic livers.

## Abbreviations

ATP: adenosine triphosphate
BCAAs: branched-chain amino acids
CV: coefficient of variation
DM: diabetes mellitus
H&E: Hematoxylin and Eosin
^1^H NMR: proton nuclear magnetic resonance spectroscopy
NAD: nicotinamide adenine dinucleotide
PC: principal component
PLS-DA: partial least squares-discriminate analysis
QC: quality control
SAM: S-adenosylmethionine
STZ: streptozotocin
TCA: tricarboxylic acid
TSP: trimethylsilyl-propionic-2,2,3,3d4-acid
T1DM: type 1 DM
T2DM: type 2 DM
